# High Buildings and Elevators in New York

**Published:** 1881-10

**Authors:** 


### HIGH BUILDINGS AND ELEVATORS IN NEW YORK.

An increasingly characteristic feature of the business portion of New York is its lofty buildings for offices. Of the score of office buildings now going up or nearly finished there is only one—the Stock Exchange—which is less than twelve stories high. The Stock Exchange is only four stories high, it is said, for the reason that if it had been carried higher and the upper floors rented to brokers, the competition would have been so great for these offices that ill-feeling would have been engendered.

Recently a journalist had occasion to make a dozen business calls in this part of the city, and out of curiosity kept a record of the height he traveled in elevators. He says :

"For eleven of the twelve calls I had to enter an elevator, and twice I retraced my steps, finding my man out the first time. Adding up the number of stories I was lifted, I find that I went up sixty-two stories, or a total height of 806 feet, allowing an average of thirteen feet to each story—a very small average. This is nearly twice the height of the Great Pyramid of Egypt, and any traveler who goes to the top of the Great Pyramid in less than half an hour on a hot day will be able to estimate the saving in strength effected by our New York elevators. If all our elevators were to break down at once business would come to a standstill.—*Scientific American.*

